# Critical Involvement of Glial Cells in Manganese Neurotoxicity

**DOI:** 10.1155/2021/1596185

**Published:** 2021-10-06

**Authors:** Jazmín Soto-Verdugo, Arturo Ortega

**Affiliations:** Departamento de Toxicología, Centro de Investigación y de Estudios Avanzados del Instituto Politécnico Nacional, 07360 Ciudad de México, Mexico

## Abstract

Over the years, most of the research concerning manganese exposure was restricted to the toxicity of neuronal cells. Manganese is an essential trace element that in high doses exerts neurotoxic effects. However, in the last two decades, efforts have shifted toward a more comprehensive approach that takes into account the involvement of glial cells in the development of neurotoxicity as a brain insult. Glial cells provide structural, trophic, and metabolic support to neurons. Nevertheless, these cells play an active role in adult neurogenesis, regulation of synaptogenesis, and synaptic plasticity. Disturbances in glial cell function can lead to neurological disorders, including neurodegenerative diseases. This review highlights the pivotal role that glial cells have in manganese-induced neurotoxicity as well as the most sounding mechanisms involved in the development of this phenomenon.

## 1. Introduction

The central nervous system (CNS) is comprised mainly of two types of cells: neurons and glia. From a neurocentric point of view, neurons are the cells that process and transfer information in the brain by triggering action potentials and propagating these electrical signals [1]. On the other hand, glial cells, which are as numerous as neurons across the whole brain [2], were first described as the connective structure that holds nerve cells in place. However, in the last decades, these cells are starting to be recognized as master regulators of synaptic plasticity [3]. Glial cells can be classified into two main categories: macroglia and microglia. Oligodendrocytes and astrocytes (members of the former category) are originated in the embryonic neural tube and forebrain from neural progenitor cells (NPC), then NPCs are transformed into radial glia, which are precursors of neurons and glia [4]. Following the production of neurons, a “gliogenic switch” allows radial glia to give rise to astrocytes and oligodendrocytes. Oligodendrocytes, the myelinating glia of the CNS, are produced *via* the generation of intermediate precursor cells known as NG2 glia or oligodendrocyte precursor cells (OPC). Astrocytes are also produced from radial glia but in response to distinct external signals in specific regions of the brain. Interestingly, microglia are generated from primitive macrophages in the embryonic yolk sac that migrates to the CNS and becomes microglia [4, 5]. Each of these types of glial cells has essential roles in CNS development and is a fundamental piece for the correct functioning of the brain.

Manganese (Mn) is the 5^th^ and 12^th^ more abundant metal and element, respectively; it is ubiquitously found across the earth's crust. Moreover, Mn is an essential trace element required for proper physiological development and tight regulation of cellular and biochemical reactions [6, 7]. This element is mainly found in its Mn^2+^ and Mn^3+^ species within mammalian tissues, although it can be found in a great variety of oxidation states [6]. Due to their essentiality, Mn is an important cofactor of enzymes like glutamine synthetase (GS), superoxide dehydrogenase (SOD), arginase, and pyruvate carboxylase. Some of the main functions in which Mn has been implicated are protein, lipid, and carbohydrate metabolism, detoxification of reactive oxide species (ROS), immune response, energy metabolism, and glucose regulation, among others [8, 9]. Nevertheless, chronic overexposure to this transition metal may result in a neurological disorder known as “manganism,” which resembles some of the symptoms of Parkinson's disease (PD) [10]. The toxic effects of Mn in the brain respond to an increase in the levels of this metal by around three times the concentration found in “normal” conditions. The “normal” concentrations are ranging from 1.1-2.9 ppm in the whole human brain [11]. Hence, Mn is necessary for proper brain function, yet alterations in its physiological levels can cause neurotoxic effects, either overexposure or insufficiency, although the last one is less common [12], which points out the hormetic nature of Mn and its nonmonotonic dose-response patterns for the development of neurotoxicity. The main routes of exposure are inhalation due to exposure to Mn enriched dust, fumes, or particulate matter and by ingestion of food or water rich in Mn. Upon Mn absorption, this metal widely distributes to a variety of body compartments [13]. The capability of Mn to cross several blood-tissue barriers resides in its potential to be transported by several membrane carriers. The divalent metal transporter 1 (DMT1), transferrin receptor (TfR), zinc transporters ZIP8 and ZIP14, citrate transporter, choline transporter, dopamine transporter (DAT), and calcium (Ca^2+^) channels had been described for import of this metal, whereas ferroportin (Fpn), SLC30A10, ATPase 13A2 (ATP13A2), and secretory pathway Ca^2+^-ATPase 1 (SPCA1) for the export [14]. Mn is eliminated mainly via the hepatobiliary system; meanwhile, other excretion pathways like the urinary and pancreatic are minimally engaged in Mn clearance [15]. In humans, the whole-body half-life of Mn has been described after oral administration and intravenous injection, with values ranging from 6-43 days and 24-74 days, respectively [16]. However, loss of function in Mn transporters promotes its accumulation in the CNS. Hepatic dysfunction, such as cirrhosis and hepatic encephalopathy, increases the risk of excessive build-up of Mn concentrations in the brain [17].

This contribution is aimed at emphasizing the pivotal role that glial cells have in the neurotoxicity induced by Mn exposure and the most supported mechanisms involved in the development of this phenomenon.

## 2. The Importance of Glial Cells in Neurotoxicity Development

The days when glial cells were considered the “glue” of synapses are fortunately long past gone. Nowadays, increasing evidence has emphasized their crucial role in proper brain functioning and CNS development [18]. Indeed, these cells provide structural, trophic, and metabolic support to neurons [19] and play an active role in important brain functions such as the uptake and synthesis of neurotransmitters, buffering of ion strength, immunomodulation, and adult neurogenesis, acting as a part of the blood-brain barrier controlling the in and out of substances from the bloodstream to the brain, intercellular communication through the formation of a glial syncytium, regulation of synaptogenesis, and synaptic plasticity while associated to synapses [4, 20]. Even though glial cells are incapable of firing action potentials, these cells can communicate with other cells through chemical signals, such as neurotransmitters, ions, neurotrophic, and neurotoxic factors; since these cells express a broad repertoire of membrane transporters, neurotransmitters as well as neurotrophic receptors, voltage-gated ion channels, and ion exchangers that allow them to receive and send signals to the neurons and other glia [21, 22]. Within the glial network, calcium activity (spatial and temporally coordinated) influences different states of the neuronal network leading to repercussions in high-order cognitive functions [23]. Over the last years, it has been well documented that disturbances in glia physiology can lead to neurological disorders, including neurodegenerative diseases such as PD, Alzheimer's, and Huntington's diseases (AD and HD), as well as epilepsy, ischemic stroke, depression, autism, or glioma [1]. This shifting from the neurocentric approach has spotlighted the fact that there is more nuance in glial cells' crosstalk with neurons than previously thought. Moreover, whether glia dysfunction is the cause or a consequence of neurotoxicology development is a question that remains to be determined but is undoubtedly glia is a key player that should not be neglected.

### 2.1. Glia Involvement in Mn Neurotoxicity

Several studies have focused on the effects of Mn toxicity in the CNS since James Couper first described *“*manganism” as a neurological disorder caused by overexposure to Mn dioxide in the midnineteenth century [24]. Over the years, most of the research concerning Mn exposure was centered on the disruption of neurons, given that these cells are more sensitive to the toxic effects of Mn. However, in the last two decades, it has shifted to a more comprehensive approach that considers the involvement of glia in the development of neurotoxicity [25, 26]. Some of the major effects of Mn toxicity in glia are listed below.

#### 2.1.1. Astrocytes

The most abundant type of glial cells in the CNS is astrocytes. A single human astrocyte is capable of ensheathing more than 100,000 synapses [5]. The best-characterized functions of these cells are the turnover of neurotransmitters, ion homeostasis, as a constituent of the neurovascular unit, synaptogenesis, neuronal remodeling, and so on [18]. Astrocytes can be classified at least into two main categories regarding their localization and morphology: fibrous in the white matter with a clear star shape, and protoplasmic in the grey matter with a more plain appearance [27]. Furthermore, astrocytes oversee and protect neurons from neurotoxic insults elicited by heavy metals, which makes them the primary target of heavy metal toxicity [28]. Mn accumulates 50-200 times more in astrocytes than in neurons [29, 30]. Besides, astrocytes are more resilient to the cytotoxic effects of Mn exposure [31, 32], but even at concentrations below the cytotoxic threshold, there are several adverse effects in glial cells that could affect glia-neuron homeostasis. The first study that put in the limelight the repercussions of Mn overexposure on astrocytes observed that Mn increases nitric oxide (NO) synthesis in astrocytes [33]. NO is a free radical and a known signaling messenger that may cause detrimental consequences to neighboring neurons when it is produced persistently [34]. Several studies have demonstrated that Mn exposure disrupts the glutamate/glutamine cycle (GGC) [35], rendering a myriad of adverse consequences that are going to be discussed in further detail below.

#### 2.1.2. Radial Glia

As mentioned before, radial glia is a specialized type of astrocyte that has been identified as a primary progenitor cell for both astrocytes and neurons. Radial glia serves as a scaffold for the migration of nascent cortical and cerebellar neurons, underlining their pivotal role in CNS development [21]. Moreover, these cells have an active role in adult neurogenesis due to their proven capacity to divide and give rise to new neurons, in response to brain injury, acting as neural stem cells in the adult CNS [5]. There is a great diversity of radial glia in the CNS; Müller radial glia can be found across the retina, in the cerebellum: Bergmann glia, tanycytes are located in the third ventricle of the brain, to mention few examples [36]. An increase in the catalytic efficiency of glutamate transporters, as well as a decrease in glucose transport of Bergmann glia, was found after short-term exposure to Mn, with an absence of cell death [37]. This is not a minor finding since glial metabolism is predominantly glycolytic and metabolic coupling of glutamate and glucose transport has been described [38]. Considering that Bergmann glia outnumbers neurons in the cerebellar cortex and that the neurotoxic effects of Mn over cerebellar granular neurons are more pronounced than in neocortical neurons [39], the potential role of the local radial glia in the neurotoxic effects of Mn should not be taken lightly.

#### 2.1.3. Microglia

Widely known as the resident immune cell of the CNS, microglia makes up about 10% of the CNS glia [22]. The leading roles of microglia are immune surveillance, extracellular matrix remodeling, clearance of cellular debris and synaptic pruning, neurogenesis regulation, among other functions [18, 22]. Under basal conditions, microglia have a distinct ramified morphology with extended processes for CNS surveillance. Meanwhile, upon a pathologic scenario, these cells present enlarged somas and sprouts and an ameboid or hypertrophic phenotype [40]. In an activated mode, microglia triggers and maintains an inflammatory response, deluging neurons to inflammatory mediators leading to neuronal cell death, making them an essential mediator of neurotoxicity phenomena. Mn exposure upregulates inducible nitric oxide synthase (iNOS) and tumor necrosis factor-*α* (TNF-*α*), and interleukin-1*β* (IL-1*β*) with dopaminergic dysfunction after microglial activation [41, 42]. Moreover, microglial TNF-*α* and IL-1*β* release induced by Mn is presumably triggered by the activation of the Janus kinase 2/signal transducer and activator of transcription 3 (JAK2-STAT3) signaling pathway [43]. Sodium *para*-aminosalicylic acid (PAS-Na) prevents Mn effects [44, 45]. Moreover, Mn treatment induces microglial cell death by regulating necrosis due to lysosomal membrane permeabilization and cathepsin activation [46].

## 3. Mechanisms of Toxicity Elicited by Mn in Glial Cells

### 3.1. Glu/Gln Cycle

**Figure 1:**
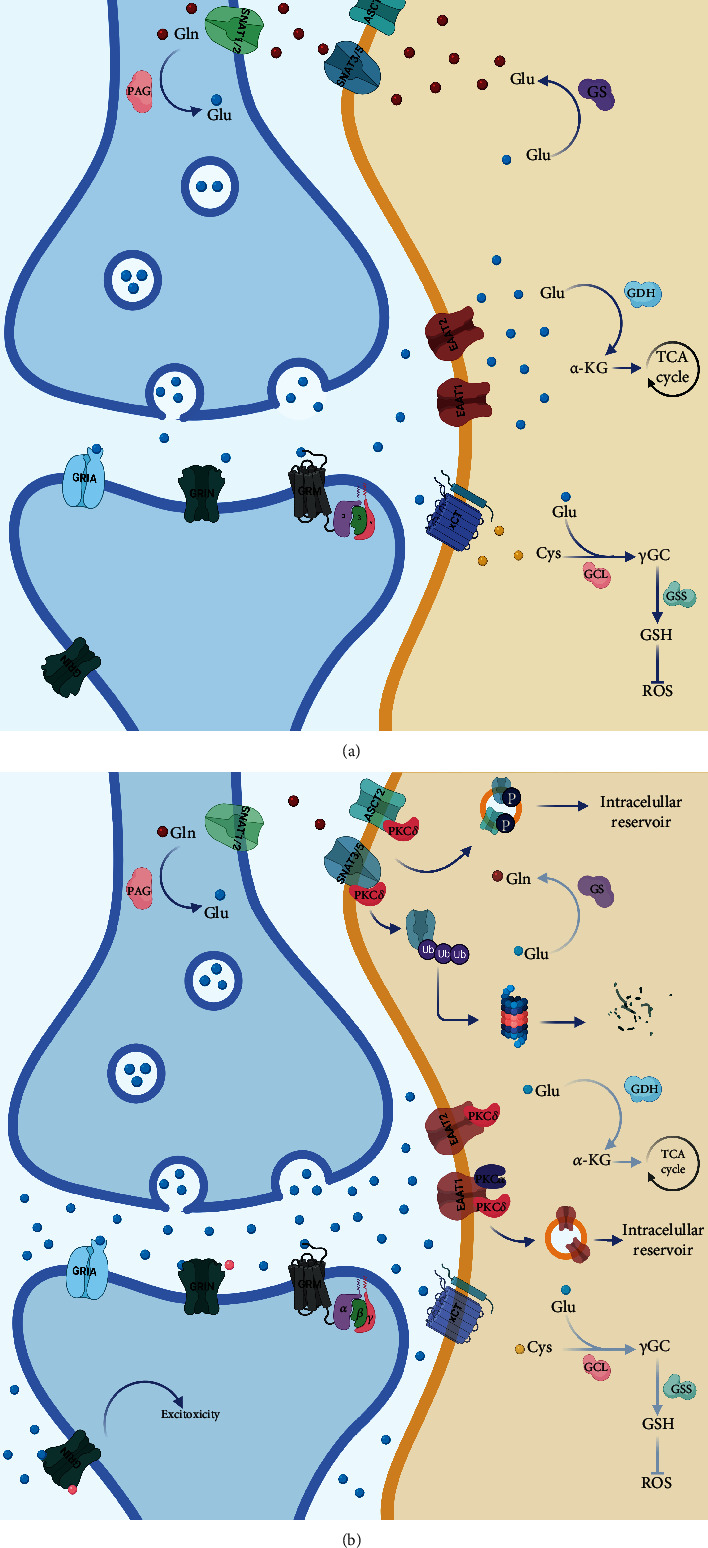
Effect of Mn exposure over the glutamatergic tripartite synapse. (a) Glu/Glu shuttle in normal conditions; Glu levels at the synaptic cleft are tightly regulated by EAATs in glial cells, once inside Glu can be transformed to Gln by GS and then Gln transported by SNATs to the neurons to replenish the Glu stores since Gln can be transformed to Glu by glutaminase. (b) Mn exposure affects the main effector proteins of the Glu/Gln shuttle, when Mn has surpassed the physiological threshold, and Glu tends to accumulate in the synaptic cleft due to the downregulation of EAAT1/2, mostly due to PKC activation. This promotes the overactivation of Glu receptors, even the ones in the extra-synaptic space, triggering the activation of death signaling pathways, a phenomenon known as excitotoxic death. Moreover, GS activity is also diminished along with the downregulation of Gln transporters, distressing all levels of the Glu/Gln cycle rendering defective glutamatergic neurotransmission.

Glutamate (Glu) is the main excitatory amino acid neurotransmitter in vertebrates. Once released in the synaptic cleft exerts its actions through the activation of specific receptors expressed in the plasma membrane of neurons and glial cells. Glu receptors have been categorized into two main groups: ionotropic Glu receptors, which are ligand-gated ion channels rendering excitatory Glu-evoked currents, and metabotropic Glu receptors, which are G-protein coupled receptors managing cellular processes *via* second messenger signaling [47]. Overactivation of Glu receptors may end in neuronal death, a phenomenon coined as “*excitotoxicity”* [48]. Since no known enzyme degrades Glu in the extracellular space, the removal of Glu from the synaptic cleft is needed to maintain the levels necessary for appropriate synaptic transmission. Such work is carried out by high-affinity Na^+^-dependent transporters mainly found in astrocytes. Once inside the glial cell, the enzyme glutamine synthetase (GS) converts Glu to glutamine (Gln) or is taken up into the Krebs cycle after being transformed into *α*-ketoglutarate [49]. The transformed Gln is imported into the astrocyte by a series of Gln transporters allowing the recycling of neurotransmitters and reducing the energy expenditure of the neurons [50]. This process is termed GGC or Glu/Gln shuttle (Figure 1(a)), and the impairment of this cycle is a common mechanism of neurodegenerative diseases and other disorders [51].

#### 3.1.1. Glutamate Transport

The levels of Glu in the synaptic cleft are tightly regulated by a family of sodium-dependent plasma membrane transporters, known as excitatory amino acid transporters (EAATs) [51, 52]. There are five members in this family of transporters, glutamate/aspartate transporter (GLAST)/EAAT1, Glu transporter 1 (Glt-1)/EAAT2, excitatory amino acid carrier 1 (EAAC1)/EAAT3, EAAT4, and EAAT5. Glt-1 and GLAST are mainly expressed in astrocytes, although Glt-1 can be expressed in hippocampal neurons. EAAC1 and EAAT4 can be found mostly in neurons, whereas EAAT5 is expressed in bipolar cells and photoreceptors in the retina [53]. EAATs transport a single molecule of Glu paired with three Na^+^ ions and a proton, with the antiport of a K^+^ ion. The timely removal of Glu from the synaptic cleft and its consequent recycling is fundamental for proper glutamatergic neurotransmission and to avoid excitotoxicity (Figure 1(a)) [53]. The disruption of the Glu transport has been a focal point in the study of the critical role of glial cells in Mn neurotoxicity (Figure 1(b)). As shown in Table 1, Mn exposure disrupts glutamate transport in different models *in vivo* and *in vitro*. Glu uptake decreased in primary cortical astrocytes exposed to Mn [54] and in Chinese hamster ovarian cells transfected with GLAST and Glt-1 [55]. Nonhuman primates presented a decrease in the protein and mRNA levels of GLAST and Glt-1 in different brain regions after Mn exposure [52, 56]. Contrastingly, short-term exposure to Mn in Bergmann glia showed an increase in the uptake and catalytic efficiency of GLAST [37]. While several studies have demonstrated that Mn affects Glu transporters (Table 1), current research has been directed to dissect the mechanisms by which Mn downregulates GLAST and Glt-1. Previous reports revealed that astrocytes treated with Mn had increased activity of the protein kinase C (PKC) [57]. Besides, activation of PKC by Mn decreases Glu uptake and expression of GLAST and Glt-1. PKC*δ* isoform interacts specifically with Glt-1 while PKC*α* with GLAST (Figure 1(b)). Additionally, the lysosomal pathway appears to be responsible for the downregulation of Glu transporters [58]. Mn treatment decreases the expression of transforming growth factor-alpha and beta (TGF-*α*/*β*) [31, 59].

Recent studies demonstrate that Mn induces tumor necrosis factor-alpha (TNF-*α*) release, promoting NF-*κ*B signaling that activates the transcription factor ying-yang 1 (YY1), which along with histone deacetylases (HDACs) forms a repressor complex that decreases the levels of GLAST and Glt-1. The deletion of astrocytic YY1 attenuates the Mn-induced effect over the Glu transporter. In contrast, the interaction of YY1/HDAC with p65 overrides the stimulatory effects of NF-*κ*B over GLAST and Glt-1 promoters downregulating their expression in the plasma membrane of astrocytes [60–63]. Deletion of astrocytic YY1 attenuated the Mn-induced decrease of GLAST and Glt-1 [62]. Ephrin-A3 is known to downregulate Glu transporters; the involvement of this protein in Mn-induced downregulation of GLAST and Glt-1 seems like a plausible mechanism for Mn-elicited neurotoxicity [64]. Moreover, a special effort has been put into ameliorating the effects of Mn on Glu transporters; treatments such as raloxifene [65], arundic acid [60], valproate [66, 67], riluzole [64, 68, 69], sodium butyrate [66], 17*β*-estradiol [70], tamoxifen, fluoxetine [64], and PAS-Na [71] have proved to prevent the effects of Mn over Glu transporters.

#### 3.1.2. Glutamine Synthetase

In the brain, GS is an astrocyte-enriched protein that catalyzes the conversion of glutamate and ammonium ions into Gln, the only known source of endogenous Gln in mammals [72]. Moreover, GS is a Mn-activated enzyme that forms an octamer with four Mn^+2^ ions [73], accounting for about 80% of Mn concentration in the brain [74]. GS is essential in the recycling of neurotransmitters such as Glu and gamma-aminobutyric acid (GABA) and is crucial in ammonia detoxification and as a marker of ROS production due to its susceptibility to oxidative degradation (Figure 1(a)) [75]. Furthermore, this enzyme plays a major role in CNS function, and its disruption is linked to Alzheimer's disease incidence, temporal lobe epilepsy, schizophrenia, and other neurological disorders [76]. Chronic Mn overload has been shown to downregulate the expression and activity of GS in different *in vivo* and *in vitro* models (Figure 1(b)), such as nonhuman primates [52, 56, 77] and rodents, even at *in utero* exposure [78, 79]. Mn-induced alterations in the mRNA and protein levels of GS were also observed in primary cortical astrocytes, as well as decreased enzymatic activity (Table 2) [69, 80].

#### 3.1.3. Glutamine Transport

Gln, the most abundant amino acid in the CNS, has a pivotal role in brain metabolism and as a precursor of neurotransmitters. The transport of Gln involves the efflux from astrocytes and the consequent influx into neurons (Figure 1(a)); such a process requires a variety of transport systems [81]. Briefly, the release of Gln is mainly done by system N: sodium-coupled neutral amino acid transporter (SNAT) 3/5, although the system ASC: Alanine-Serine-Cysteine transporter (ASCT) 1/2 can also take on this duty to a lesser extent. On the other hand, the uptake can also be achieved by the systems mentioned above in addition to system A: SNAT1/2 and L: L-type amino acid transporter (LAT) 1/2; the latter pair is mostly expressed in neurons, although all transporters are expressed in glia [82]. Exposure to high Mn concentrations inhibited the uptake of Gln by cortical astrocytes in a concentration-dependent fashion and decreased the mRNA levels of SNAT1 and SNAT3 [83]. Moreover, the involvement of systems N, ASC, and L in the diminished uptake of Gln after Mn overexposure was suggested, concomitant with a drop in the mRNA and protein levels of Gln transport systems (Table 3). The decline in SNAT3 levels was associated with the transporter's degradation *via* the ubiquitin-mediated proteolytic system through the interaction of SNAT3 with the ubiquitin ligase Nedd4-2 (neural precursor cells expressed developmentally downregulated 4-2) [84]. In the same vein, Mn-induced PKC signaling has been involved in the downregulation of Gln transport (Figure 1(b)). The inhibition of PKC activation reverses the Mn-induced decrease in SNAT3-dependent Gln transport [57]. PKC*δ* isoforms bind to SNAT3 or ASCT2, possibly inducing their phosphorylation and internalization (Figure 1(b)), as previously suggested [85].

### 3.2. Mitochondrial Impairment and Energy Metabolism

**Figure 2:**
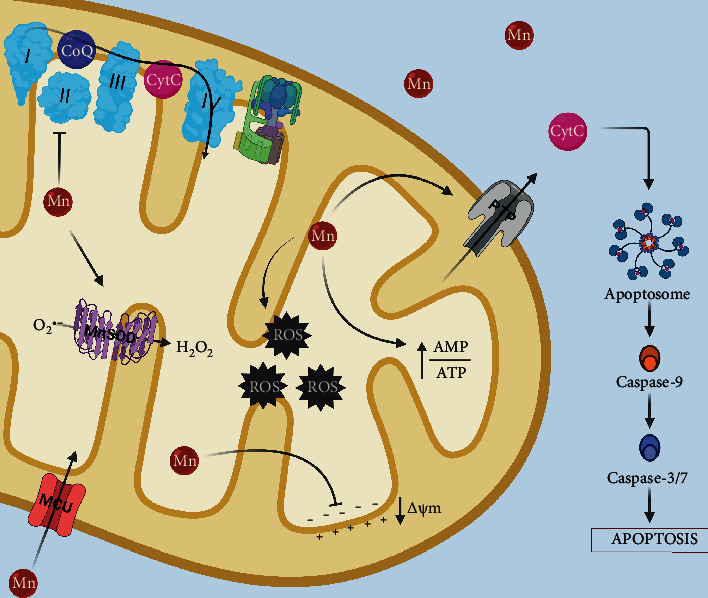
Effects of Mn exposure on mitochondrial function in glia. Once inside the cell, Mn is readily taken up by the mitochondria through the MCU, where exerts its toxic actions by producing free radicals and damaging the complex II of the electron transport chain, the excessive levels of Mn in the mitochondria can also affect Mn-SOD activity promoting hydrogen peroxide formation. Moreover, Mn depolarizes the mitochondrial membrane potential promoting the opening of the PTP, which allows the release of cytochrome C, triggering caspase-dependent apoptosis pathways.

Mn accumulates preferentially in the mitochondria through the mitochondrial Ca^2+^ uniport (MCU) [86]. Once inside, Mn exerts its toxic traits by inhibiting the oxidative phosphorylation process, sequestering Ca^2+^ in the matrix, and promoting ROS production [86, 87]. Mn treatment disrupts the energy metabolism in glial cells [29]. The treatment of cortical astrocytes with Mn induces the mitochondrial permeability transition pore (PTP), a Ca^2+^ dependant process that promotes membrane permeability (Figure 2). This leads to the disruption of the inner membrane potential, resulting in mitochondrial failure [88]. Besides, the activation of the mitogen-activated kinase (MAPK) an extracellular signal-regulated kinase (ERK) pathway induced by Mn exposure in astrocytes and the collapse of the mitochondrial membrane potential triggers apoptosis through caspase-3 activation [89]. Mn-induced apoptosis in astrocytes activates in response to depolarization of the mitochondrial membrane, which releases cytochrome C, induces caspases 3/7, and modulates the expression of B-cell lymphome 2 (Bcl-2) proteins [90]. Complex II of the respiratory chain is altered by Mn exposure, producing ROS in microglia (Figure 2) [91]. *Noteworthy, mitochondria dysfunction has been associated with inhibition of alternative activation of microglia, consequently exacerbating neuroinflammation [*93*]*. Mn exposure produces lysosomal membrane permeabilization and cathepsin release, which activates BH3-interacting domain death agonist (Bid) promoting mitochondrial damage in glial cells [93]; similar outcomes were found in microglia [46]. Human astrocytes treated with Mn presented activation of the caspase-dependent mitochondrial apoptotic pathway coupled with dysregulation of the expression levels of mitochondria-shaped proteins like mitochondrial dynamin-like GTPase (Opa-1), mitofusin 2 (Mfn-2), and dynamin-related protein 1 (Drp-1) [94].

### 3.3. Oxidative Stress

**Figure 3:**
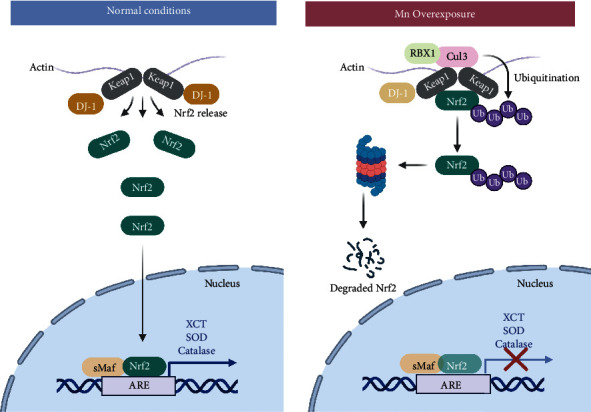
The role of Nrf2 in Mn-induced oxidative stress in glia. Nrf2 is a known regulator of the antioxidant response that translocates to the nucleus to act as a transcription factor binding to the antioxidant response element (ARE), enhancing the expression of antioxidant enzyme genes. For this to happen, it is necessary the release of Nrf2 from Keap1, such action is regulated by several effector proteins, DJ-1 is one of them. DJ-1 is downregulated in conditions of Mn overexposure, leading to the binding of Nrf2 to Keap1, promoting the degradation of Nrf2 by the ubiquitin proteasome impairing the expression of several antioxidant genes.

The main production site of ROS in the cell is the electron transport chain in the mitochondria, and as was briefly discussed before, mitochondria are a primary target of Mn toxicity, promoting ROS production [95]. In comparison to neurons, glial cells are more equipped to endure oxidative damage [96]. Once inside the cell, divalent Mn can be oxidized by ceruloplasmin to its trivalent state, known to be more toxic [97]. Proof of this is that trivalent Mn oxidizes catecholamines *via* oxidative stress [98]. Moreover, in primary cultures of astrocytes, Mn exposure increases the levels of ROS [80]. Mn-induced ROS production in the mitochondria augments nitric oxide synthase expression and activation of NF-*κ*B [99]. In addition, the nuclear factor erythroid 2-related factor (Nrf2), a known regulator of the antioxidant response (Figure 3(a)), is significantly increased in astrocytes after Mn exposure. However, at the same time, Mn reduces protein deglycase 1 (DJ-1)/PARK7 expression, a multifunctional protein that acts as a redox sensor (Figure 3(b)) [100]. This protein impairs the binding of Kelch-like ECH-associated protein 1 (Keap1) to Nrf2, avoiding its degradation by the ubiquitin proteosome and promoting Nrf2 activation allowing the transcription of several antioxidant genes (Figure 3(a)). DJ-1/PARK7 downregulation, as with Mn exposure, makes astrocytes more susceptible to oxidative stress (Figure 3(b)) [101, 102]. Another protein involved in the regulation of antioxidant genes and that also has been tied to Mn toxicity in glial cells is the forkhead box transcription factor class O (FoxO) along with the PPAR gamma coactivator-1 (PGC-1) [103]. The Mn-induced induction of oxidative stress proved to impair the ability of astrocytes to promote axonal and neurite outgrowth [104]. Recently, it has been shown that Mn alters glutathione (GSH) synthesis by inhibiting the glutamate/cystine antiporter (xCT) due to the induction of oxidative stress in striatum astrocytes [105].

### 3.4. Calcium Homeostasis

An accumulating body of evidence indicates that dysregulation of calcium (Ca^2+^) homeostasis is closely related to several neurodegenerative diseases, psychiatric disorders, and neurotoxic insults [106]. Even though glial cells do not fire action potentials, they are excitable in terms of intracellular signaling. Ca^2+^ is an important second messenger that has a great variety of cellular functions. Notably, in astrocytes, neurotransmitters activate Ca^2+^ signaling regulating glial processes such as energy expenditure *and* synaptic plasticity [107]. *D*ivalent metal cations tend to mimic some of the activities of Ca^2+^, *and* Mn is one of these metals capable of competing for certain binding sites of Ca^2+^ as well for transport systems, which makes it plausible that Mn interfere*s* in Ca^2+^ regulation [108]. The exposure of astrocytes to Mn results in the sequestering of Ca^2+^ within the mitochondria decreasing the available pool of releasable Ca^2+^ from the endoplasmic reticulum (ER), ending with the inhibition of intercellular Ca^2+^ waves, which are essential for purinergic signaling in astrocytes [109]. In the same line of study, the inhibition of ATP-induced Ca^2+^ waves and transients by Mn was mediated by the Ca^2+^ entry via the transient receptor potential channel (TRPC3) in striatal astrocytes [110]. Recently, it has been demonstrated that astrocytes transfer functional mitochondria to the neurons in a Ca^2+^-dependent manner during neuronal damage [111]. Moreover, several studies have described how Glu transport is coupled to Ca^2+^ influx through the Na^+^/Ca^2+^ exchanger (NCX) [112]. These studies, in conjunction with the ones concerning Mn toxicity [108], demonstrate the crucial role of Ca^2+^ dysregulation in glial cells after Mn exposure.

### 3.5. Autophagy

**Figure 4:**
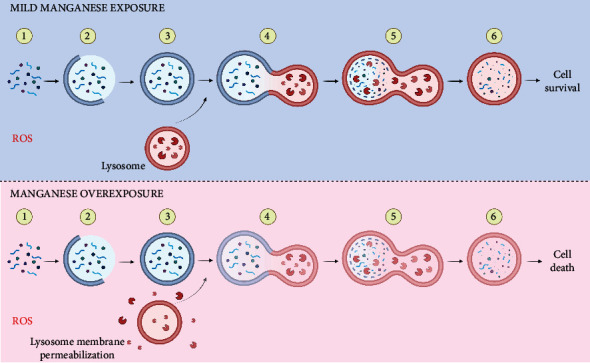
Effect of Mn exposure on autophagy in glia. Mn exposure induces ROS promoting organelle and protein damage (1). Under a “mild” Mn insult, glial cells increase their autophagic flux by increasing the expression of proteins such as Beclin1, LC3-II, and p62, the increase in these proteins would promote the initiation of the phagophore (2) and consequently the autophagosome (3). Then, after the fusion of the autophagosome with the lysosome (4), the autolysosome is created (5), allowing the degradation and recycling of damaged cellular components (6), to ameliorate Mn damage promoting cell survival. However, continuous overexposure to Mn disrupts this process by inducing lysosomal membrane permeabilization with the consequent release of cathepsins to the cytosol, leading to autophagosome accumulation and a truncated autophagic flux ending in cell death.

In recent years, the role of autophagy in the context of Mn toxicity has attracted some attention [113]. Autophagy, which means self-eating in the Greek language, is an essential mechanism for the degradation of damaged subcellular components or protein aggregates. It is a highly regulated process consisting of several steps that could be summarized as the engulfment of bulk cytoplasm forming a double-membrane vacuole, namely, “autophagosome.” Then, it is transported and fused to the lysosomes comprising the autolysosome, where finally, the degradation process takes place [114]. Either overactivation or suppression of autophagy can be involved in the pathogenesis of several neurodegenerative diseases [115]. Regarding the effects of Mn exposure in the process of autophagy, it has been reported, in *in vitro* as well as *in vivo* models, that upon short-term Mn overexposure, there is an increase in the expression of proteins such as Beclin1, microtubule-associated protein 1 light chain 3 (LC3-II), and p62 which lead to the activation of autophagy as a mechanism of coping with the Mn insult. However, as the exposure to Mn progresses, the damage produced by this metal intensifies, suppressing the autophagic process triggering neuronal death [113]. Although most of these effects have been described in neuronal models, few studies have focused on the glia component concerning the role of autophagy in Mn neurodegeneration. The first study made in glia revealed that after Mn exposure, autophagy was activated to alleviate the toxic effects of this metal (Figure 4(a)) [116]. Moreover, this effect could be mediated, at least in part by heme oxygenase-1 (HO-1) [117]. In contrast, in a primary astrocyte culture, exposure to Mn decreased the autophagic influx by inhibiting transcription factor EB (TFEB) activity [118], since active TFEB leads to a global enhancement of lysosomal catabolic efficiency [119]. In microglia, it has also been described that Mn disrupts autophagy. It was demonstrated that through the Mn-induced upregulation of leucine-rich repeat kinase 2 (LRRK2), autophagy-related proteins were dysregulated and inflammation increased [120]. Furthermore, the disruption of the autophagic process by Mn leads to NLR family, pyrin domain containing 3- (NLRP3-) caspase 1 inflammasome activation and the release of interleukin-1*β* (IL-1*β*) [121], which is associated with declined autophagic capacity. Taken together, dysregulation of autophagy seems to ameliorate Mn cytotoxic effects by increasing the autophagic flux, as shown in BV-2 cells exposed to Mn, where a time-dependent increase in the expression of LC3-II and p62, delaying Mn cytotoxicity, however, prolonged exposure to Mn increases the amount of ROS inducing lysosomal alterations (Figure 4(b)), such as lysosomal membrane permeabilization (LMP) due to the presence of the proteolytic cleavage products of poly (ADP-ribose) polymerase 1 (PARP1) [46], promoting the release of cathepsins, leading to autophagosome accumulation and ultimately cell death (Figure 4(b)) [122].

### 3.6. Neuroinflammation

The first reports regarding Mn toxic effects in glial cells were related to the release of inflammatory mediators due to glia activation [33, 123] and remain one of the principal mechanisms of Mn-mediated toxicity. Glial cell activation is known as the hallmark of neuroinflammation in addition to peripheral immune cells and the release of proinflammatory mediators [124]. Mn-induced glial activation promotes gliosis in the basal ganglia [26], which increases neuronal damage, promoting the progression of the neurotoxicological disorder. Furthermore, exposure to Mn releases inflammatory intermediaries that activate reactive astrocytes [99, 125]. In this regard, nuclear factor kappa B (NF-*κ*B) signaling in astrocytes has been implicated in the neuroinflammatory effects of Mn exposure. LRRK2 has also been implicated in Mn-induced microglia activation and consequent neuroinflammation [120, 126]. Mn exposure stimulates microglia to release hydrogen peroxide with the downstream activation of MAPK [127] and activation of NF-*κ*B signaling, promoting inflammatory responses by regulating cytokines and chemokines that amplify astrocytes' activation [128]. In addition, Mn also increases JAK2/STAT3 signaling in microglia increasing neuronal death due to the release of TNF-*α* and IL-1*β* [43]. The NLRP3 inflammasome pathway has been suggested to play a critical role in Mn-induced neuroinflammation [121, 129, 130]. Moreover, Mn exposure induces aggregation of *α*-synuclein-induced inflammation in astrocytes, impairing mitochondrial bioenergetics [131]. Further studies that take into consideration glial cells crosstalk during neuroinflammation are needed since it has been reported that activated microglia induce neurotic reactive astrocytes after acute CNS injury [132, 133].

## 4. Conclusion

A significant effort has been directed to dissect the functional and molecular events that Mn triggers in neurotoxicity development, but always taking the neuronal component as the main actor. Meanwhile, glia has always been relegated to a mere supporting role for neurons. In this contribution, we focused on the glial component as an important target of Mn deleterious effects. We provide herein an overview of the role of glial cells in Mn neurotoxicity, including the consequences on energy metabolism, redox homeostasis, Ca^2+^ signaling, inflammation, and autophagia. Moreover, we detail the key findings in the Mn-induced disruption of the Glu/Gln shuttle.

Still, many questions remain to be answered regarding the role of glia in Mn neurotoxicity; the evidence so far demonstrates that these cells have a pivotal role in the management of the Mn insult and its relevance to the neuronal counterpart. It is time to reevaluate Mn neurotoxicity as a whole and consider both neurons and glia as targets. Further investigations are needed to take into consideration the close relationship that neurons and glia maintain. Cocultures of glial cells in physical contact with neurons or separated by a semipermeable membrane barrier would allow us to link the individual effects that had already been described in each cellular model and shed light on this health problem.

## Figures and Tables

**Table 1:** Effect of Mn exposure on glutamate transporter expression and activity *in vitro* and *in vivo*.

Model	Treatments		EAAT1/GLAST			EAAT2/GLT-1			Ref.
	[Mn]	T	mRNA	Protein	Activity	mRNA	Protein	Activity	
Rat cortical astrocytes	MnCl_2_ 100 *μ*M	2 d	↓ activity in Glu uptake in general						[54]
Rat cortical astrocytes	MnCl_2_ 250-500 *μ*M	≈18 h	↓	−	↓	−	−	−	[134]
Rat cortical astrocytes	MnCl_2_, MnPO_4_, and MnSO_4_ 100-300 *μ*M	6 h	↓	−	−	−	−	−	[135]
DbB7 cell line	MnCl_2_ 0.5-1 mM	6 h	−	−	↓	−	−	↓	[55]
Rhesus monkey	0.18, 0.92, and 4.62 mg MnSO_4_/m^3^	65 d	↑ (GP,Cb)	↓ (GP,Cb,OC, FC)	−	↓ (C,GP,OC)	↓ (C,GP,Cb,OC)	−	[52]
Rhesus monkey	1.5 mg MnSO_4_/m^3^	15-65 d	↓ (Cb) ↑ (GP,OC)	↓ (GP)	−	↑ (C, Cb,FC)	↓ (GP,OC)	−	[56]
Rat cortical astrocytes	MnCl_2_ 250-500 *μ*M	6 h	↓	↓	↓	↓	↓	↓	[27, 40, 41, 51, 53, 56]
Rat striatum	8, 40, and 200 *μ*M/kg MnCl_2_	4 w	↓	↓	−	↓	↓	−	[68]
Rat cortical astrocytes	MnCl_2_ 250-500 *μ*M	24 h	↓	↓	↓	↓	↓	↓	[69]
Rat cortical astrocytes	MnCl_2_ 100 and 500 *μ*M	0.5-24 h	−	↓	↓	−	↓	↓	[58]
Mouse cortex and Cb	30 mg/kg MnCl_2_	21 d	↓	↓	−	↓	↓	−	[66]
H4 cell line and mouse brain	250 *μ*M; 30 mg/kg MnCl_2_	6 h; 21 d	↓	↓	−	↓	↓	−	[67]
Mouse St and Cb	1 *μ*mol/*μ*l of MnCl_2_	1 w	↓	↓	−	↓	↓	−	[70]
Mouse St astrocytes and St	500 *μ*M; 50 mg/kg MnCl_2_	24 h; 2 w	↓	↓	−	↓	↓	−	[64]
Chick Bergmann glia	MnCl_2_ 200 *μ*M	30'	↓ (24 h)	−	↑	−	−	−	[37]
Rat brain (St, GP, Hp, and Th)	15 mg/kg MnCl_2_	4 w	↓	−	−	↓	−	−	[71]
Mouse midbrain	30 mg/kg MnCl_2_	21 d	↓	↓	−	↓	↓	−	[62]

↑: increase; ↓: decrease; −: not analyzed; Cb: cerebellum; St: striatum; GP: globus pallidus; C: caudate; P: putamen; FC: frontal cortex; OC: olfactory cortex; Hp: hippocampus; Th: thalamus.

**Table 2:** Effect of Mn exposure on glutamine synthetase expression and enzymatic activity *in vivo* and *in vitro*.

Model	Treatments		mRNA	Protein	Activity	Ref.
	[Mn]	Time				
Sprague-Dawley rats	6 mg/kg MnCl_2_	30 d	↑	−	−	[136]
Sprague-Dawley rats	25 and 50 mg/kg MnCl_2_	PN: 21 d	−	n.s.	n.s.	[137]
Rat cortical astrocytes	MnCl_2_ 100 and 200 *μ*M	24 h	−	↑	−	[80]
Sprague-Dawley rats	0.03, 0.3, and 3 mg MnSO_4_/m^3^	14 d	↑ (Cb)	↑ (OB,Ht) ↓ (Cb)	−	[138]
Sprague-Dawley rats	0.05, 0.5, or 1 mg MnSO_4_/m^3^	13 w	↑ (Ht^F^) ↓ (Cb^M^,OB^M^,Hp^M^)	↑ (OB^F^,Hp^M^) ↓ (Hp^F^,Ht^M^)	−	[139]
Sprague-Dawley rats	0.05, 0.5, or 1 mg MnSO_4_/m^3^	IU: 19 d PN:18 d	↓	↓	−	[78, 79]
Rhesus monkey	0.18, 0.92, and 4.62 mg MnSO_4_/m^3^	65 d	↓ (FC,OC,C)	↓ (GP,Cb,FC,P)	−	[52]
Wistar rats (St, GP)	100 mM MnCl_2_	13 d	−	↓	↓	[140]
Rhesus monkeys	1.5 mg MnSO_4_/m^3^	15-65 d	↓ (C)	↓ (Cb,GP,P)	−	[56]
Sprague-Dawley rats (St)	8-200 *μ*M/kg MnCl_2_	4 w	↓	↓	↓	[68]
*Cynomolgus macaques*	3-10 mg/kg MnCl_2_	7-59 w	−	↓(GP)	−	[77]
Wistar rats	200 *μ*M/kg MnCl_2_	4 w	−	−	↓	[141]
Rat cortical astrocytes	MnCl_2_ 125-500 *μ*M	24 h	↓	↓	↓	[69]
Sprague-Dawley rats (St,GP,Th)	15 mg/kg MnCl_2_	4 w	−	−	↓	[71]

↑: increase; ↓: decrease; −: not analyzed; n.s.: no significative; Cb: cerebellum; St: striatum; GP: globus pallidus; C: caudate; P: putamen; FC: frontal cortex; OC: olfactory cortex; Hp: hippocampus; Th: thalamus; Ht: hypothalamus; IU: *in utero*; PN: postnatal; ^F^: female; ^M^: male.

**Table 3:** Effect of Mn exposure on glutamine transporter expression and activity *in vitro.*

Model	Treatments		Transporters	mRNA	Protein	Activity	Ref.
	[Mn]	Time					
Rat cortical astrocytes	MnCl_2_ 100 and 500 *μ*M	30' and 24 h	*System A*		−	↓∗	[83]
			SNAT1	↓			
			*System N*				
			SNAT3	↓			
			*System ASC*				
			ASCT2	n.s.			
Rat cortical astrocytes	MnCl_2_ 0.1, 0.5, and 1 mM	1-24 h	*System A*				[142]
			SNAT2	↓	↓	n.s.	
			*System N*				
			SNAT3	↓	↓	↓	
			*System L*				
			LAT2	↓	↓	↓	
			*System ASC*				
			ASCT2	−	**↓**	↓	
Rat cortical astrocytes	MnCl_2_ 0.1, 0.5, and 1 mM	4 h	*System N*	−	**↓**	−	[84]
			SNAT3				
Rat cortical astrocytes	MnCl_2_ 0.5 and 1 mM	4-24 h	*System A*	−			[57]
			SNAT2		n.s.	n.s.	
			*System N*				
			SNAT3		↓	↓	
			*System L*				
			LAT2		n.s.	n.s.	
			*System ASC*				
			ASCT2		↓	↓	

↑: increase; ↓: decrease; −: not analyzed; n.s.: no significative; ∗: all systems Gln uptake.

## References

[1] Maiolo L., Guarino V., Saracino E. (2021). Glial interfaces: advanced materials and devices to uncover the role of astroglial cells in brain function and dysfunction. *Advanced Healthcare Materials*.

[2] von Bartheld C. S., Bahney J., Herculano-Houzel S. (2016). The search for true numbers of neurons and glial cells in the human brain: a review of 150 years of cell counting. *The Journal of Comparative Neurology*.

[3] Wahis J., Hennes M., Arckens L., Holt M. G. (2021). Star power: the emerging role of astrocytes as neuronal partners during cortical plasticity. *Current Opinion in Neurobiology*.

[4] He F., Sun Y. E. (2007). Glial cells more than support cells?. *The International Journal of Biochemistry & Cell Biology*.

[5] Zuchero J. B., Barres B. A. (2015). Glia in mammalian development and disease. *Development*.

[6] Erikson K. M., Aschner M. (2019). 10. Manganese: its role in disease and health. *Metal Ions in Life Sciences*.

[7] Avila D. S., Puntel R. L., Aschner M. (2013). Manganese in health and disease. *Metal Ions in Life Sciences*.

[8] Wedler F. C. (1993). 3 Biological Significance of Manganese in Mammalian Systems. *Progress in Medicinal Chemistry*.

[9] Takeda A. (2003). Manganese action in brain function. *Brain Research Reviews*.

[10] Martin K. V., Edmondson D., Cecil K. M. (2020). Manganese exposure and neurologic outcomes in adult populations. *Neurologic Clinics*.

[11] Bowman A. B., Aschner M. (2014). Considerations on manganese (Mn) treatments for _in vitro_ studies. *Neurotoxicology*.

[12] Balachandran R. C., Mukhopadhyay S., McBride D. (2020). Brain manganese and the balance between essential roles and neurotoxicity. *The Journal of Biological Chemistry*.

[13] Chen P., Culbreth M., Aschner M. (2016). Exposure, epidemiology, and mechanism of the environmental toxicant manganese. *Environmental Science and Pollution Research*.

[14] Nyarko-Danquah I., Pajarillo E., Digman A., Soliman K. F. A., Aschner M., Lee E. (2020). Manganese accumulation in the brain via various transporters and its neurotoxicity mechanisms. *Molecules*.

[15] Chen P., Bornhorst J., Aschner M. (2018). Manganese metabolism in humans. *Frontiers in Bioscience*.

[16] Yokel R. A., Crossgrove J. S. (2004). Manganese toxicokinetics at the blood-brain barrier. *Research Report (Health Effects Institute)*.

[17] Chen P., Chakraborty S., Mukhopadhyay S. (2015). Manganese homeostasis in the nervous system. *Journal of Neurochemistry*.

[18] Lago-Baldaia I., Fernandes V. M., Ackerman S. D. (2020). More than mortar: glia as architects of nervous system development and disease. *Frontiers in Cell and Development Biology*.

[19] Jean Y. Y., Bagayogo I. P., Dreyfus C. F. (2009). Release of trophic factors and immune molecules from astrocytes. *Astrocytes in (Patho) Physiology of the Nervous System*.

[20] Becerra-Calixto A., Cardona-Gómez G. P. (2017). The role of astrocytes in neuroprotection after brain stroke: potential in cell therapy. *Frontiers in Molecular Neuroscience*.

[21] Stevens B. (2003). Glia: much more than the neuron's side-kick. *Current Biology*.

[22] Sancho L., Contreras M., Allen N. J. (2021). Glia as sculptors of synaptic plasticity. *Neuroscience Research*.

[23] Semyanov A. (2019). Spatiotemporal pattern of calcium activity in astrocytic network. *Cell Calcium*.

[24] Blanc P. D. (2018). The early history of manganese and the recognition of its neurotoxicity, 1837-1936. *Neurotoxicology*.

[25] Filipov N. M., Dodd C. A. (2012). Role of glial cells in manganese neurotoxicity. *Journal of Applied Toxicology*.

[26] Tjalkens R. B., Popichak K. A., Kirkley K. A. (2017). Inflammatory activation of microglia and astrocytes in manganese neurotoxicity. *Adv. Neurobiol.*.

[27] Ravi K., Paidas M. J., Saad A., Jayakumar A. R. (2021). Astrocytes in rare neurological conditions: morphological and functional considerations. *Journal of Comparative Neurology*.

[28] Li B., Xia M., Zorec R., Parpura V., Verkhratsky A. (2021). Astrocytes in heavy metal neurotoxicity and neurodegeneration. *Brain Research*.

[29] Wedler F. C., Ley B. W., Grippo A. A. (1989). Manganese (II) dynamics and distribution in glial cells cultured from chick cerebral cortex. *Neurochemical Research*.

[30] Tholey G., Ledig M., Mandel P. (1988). Concentrations of physiologically important metal ions in glial cells cultured from chick cerebral cortex. *Neurochemical Research*.

[31] Lee E.-S. Y., Sidoryk M., Jiang H., Yin Z., Aschner M. (2009). Estrogen and tamoxifen reverse manganese-induced glutamate transporter impairment in astrocytes. *Journal of Neurochemistry*.

[32] Lee E.-S. Y., Yin Z., Milatovic D., Jiang H., Aschner M. (2009). Estrogen and tamoxifen protect against Mn-induced toxicity in rat cortical primary cultures of neurons and astrocytes. *Toxicological Sciences*.

[33] Spranger M., Schwab S., Desiderato S., Bonmann E., Krieger D., Fandrey J. (1998). Manganese augments nitric oxide synthesis in murine astrocytes: a new pathogenetic mechanism in manganism?. *Experimental Neurology*.

[34] Ledo A., Lourenço C. F., Cadenas E., Barbosa R. M., Laranjinha J. (2021). The bioactivity of neuronal-derived nitric oxide in aging and neurodegeneration: switching signaling to degeneration. *Free Radical Biology & Medicine*.

[35] Sidoryk-Wegrzynowicz M., Aschner M. (2013). Manganese toxicity in the central nervous system: the glutamine/glutamate-*γ*-aminobutyric acid cycle. *Journal of Internal Medicine*.

[36] Sild M., Ruthazer E. S. (2011). Radial glia: progenitor, pathway, and partner. *The Neuroscientist*.

[37] Escalante M., Soto-Verdugo J., Hernández-Kelly L. C. (2020). GLAST activity is modified by acute manganese exposure in Bergmann glial cells. *Neurochemical Research*.

[38] Pellerin L., Magistretti P. J. (2011). Sweet sixteen for ANLS. *Journal of Cerebral Blood Flow and Metabolism*.

[39] Hernández R. B., Farina M., Espósito B. P., Souza-Pinto N. C., Barbosa F., Suñol C. (2011). Mechanisms of manganese-induced neurotoxicity in primary neuronal cultures: the role of manganese speciation and cell type. *Toxicological Sciences*.

[40] Savage J. C., Carrier M., Tremblay M. È. (2019). Morphology of microglia across contexts of health and disease. *Methods Mol. O Biologico*.

[41] Zhao F., Cai T., Liu M., Zheng G., Luo W., Chen J. (2009). Manganese induces dopaminergic neurodegeneration via microglial activation in a rat model of manganism. *Toxicological Sciences*.

[42] Park E., Chun H. S. (2017). Melatonin attenuates manganese and lipopolysaccharide-induced inflammatory activation of BV2 microglia. *Neurochemical Research*.

[43] Yin L., Dai Q., Jiang P. (2018). Manganese exposure facilitates microglial JAK2-STAT3 signaling and consequent secretion of TNF-a and IL-1*β* to promote neuronal death. *Neurotoxicology*.

[44] Peng D., Li J., Deng Y. (2020). Sodium para-aminosalicylic acid inhibits manganese-induced NLRP3 inflammasome-dependent pyroptosis by inhibiting NF-*κ*B pathway activation and oxidative stress. *Journal of Neuroinflammation*.

[45] Fang Y., Peng D., Liang Y. (2021). Sodium P-aminosalicylic acid inhibits manganese-induced neuroinflammation in BV2 microglial cells via NLRP3-CASP1 inflammasome pathway. *Biological Trace Element Research*.

[46] Porte Alcon S., Gorojod R. M., Kotler M. L. (2018). Regulated Necrosis Orchestrates Microglial Cell Death in Manganese-Induced Toxicity. *Neuroscience*.

[47] Rodríguez-Campuzano A. G., Ortega A. (2021). Glutamate transporters: critical components of glutamatergic transmission. *Neuropharmacology*.

[48] Zhou Y., Danbolt N. C. (2014). Glutamate as a neurotransmitter in the healthy brain. *Journal of Neural Transmission*.

[49] Magi S., Piccirillo S., Amoroso S., Lariccia V. (2019). Excitatory amino acid transporters (Eaats): glutamate transport and beyond. *International Journal of Molecular Sciences*.

[50] Weber B., Barros L. F. (2015). The astrocyte: powerhouse and recycling center. *Cold Spring Harbor Perspectives in Biology*.

[51] Armada-Moreira A., Gomes J. I., Pina C. C. (2020). Going the extra (synaptic) mile: excitotoxicity as the road toward neurodegenerative diseases. *Frontiers in Cellular Neuroscience*.

[52] Erikson K. M., Dorman D. C., Lash L. H., Aschner M. (2007). Manganese inhalation by rhesus monkeys is associated with brain regional changes in biomarkers of neurotoxicity. *Toxicological Sciences*.

[53] Wang J., Wang F., Mai D., Qu S. (2020). Molecular mechanisms of glutamate toxicity in Parkinson’s disease. *Frontiers in Neuroscience*.

[54] Hazell A. S., Norenberg M. D. (1997). Manganese decreases glutamate uptake in cultured astrocytes. *Neurochemical Research*.

[55] Mutkus L., Aschner J. L., Fitsanakis V., Aschner M. (2005). The in vitro uptake of glutamate in GLAST and GLT-1 transfected mutant CHO-K1 cells is inhibited by manganese. *Biological Trace Element Research*.

[56] Erikson K. M., Dorman D. C., Lash L. H., Aschner M. (2008). Duration of airborne-manganese exposure in rhesus monkeys is associated with brain regional changes in biomarkers of neurotoxicity. *Neurotoxicology*.

[57] Sidoryk-Wegrzynowicz M., Lee E., Mingwei N., Aschner M. (2011). Disruption of astrocytic glutamine turnover by manganese is mediated by the protein kinase C pathway. *Glia*.

[58] Sidoryk-Wegrzynowicz M., Lee E., Aschner M. (2012). Mechanism of Mn(II)-mediated dysregulation of glutamine-glutamate cycle: focus on glutamate turnover. *Journal of Neurochemistry*.

[59] Lee E., Sidoryk-Wegrzynowicz M., Yin Z., Webb A., Son D. S., Aschner M. (2012). Transforming growth factor-*α* mediates estrogen-induced upregulation of glutamate transporter GLT-1 in rat primary astrocytes. *Glia*.

[60] Karki P., Hong P., Johnson J. (2018). Arundic acid increases expression and function of astrocytic glutamate transporter EAAT1 via the ERK, Akt, and NF-*κ*B pathways. *Molecular Neurobiology*.

[61] Karki P., Kim C., Smith K., Son D. S., Aschner M., Lee E. (2015). Transcriptional Regulation of the Astrocytic Excitatory Amino Acid Transporter 1 (EAAT1) via NF-*κ*B and Yin Yang 1 (YY1). *The Journal of Biological Chemistry*.

[62] Pajarillo E., Johnson J., Rizor A. (2020). Astrocyte-specific deletion of the transcription factor yin yang 1 in murine substantia nigra mitigates manganese-induced dopaminergic neurotoxicity. *The Journal of Biological Chemistry*.

[63] Karki P., Webb A., Smith K. (2014). Yin yang 1 is a repressor of glutamate transporter EAAT2, and it mediates manganese-induced decrease of EAAT2 expression in astrocytes. *Molecular and Cellular Biology*.

[64] Qi Z., Yang X., Sang Y. (2020). Fluoxetine and riluzole mitigates manganese-induced disruption of glutamate transporters and excitotoxicity via ephrin-A3/GLAST-GLT-1/Glu signaling pathway in striatum of mice. *Neurotoxicity Research*.

[65] Karki P., Webb A., Zerguine A., Choi J., Son D. S., Lee E. (2014). Mechanism of raloxifene-induced upregulation of glutamate transporters in rat primary astrocytes. *Glia*.

[66] Johnson J., Pajarillo E. A. B., Taka E. (2018). Valproate and sodium butyrate attenuate manganese-decreased locomotor activity and astrocytic glutamate transporters expression in mice. *Neurotoxicology*.

[67] Johnson J., Pajarillo E., Karki P. (2018). Valproic acid attenuates manganese-induced reduction in expression of GLT-1 and GLAST with concomitant changes in murine dopaminergic neurotoxicity. *Neurotoxicology*.

[68] Deng Y., Xu Z., Xu B. (2009). The protective effect of riluzole on manganese caused disruption of glutamate- glutamine cycle in rats. *Brain Research*.

[69] Deng Y., Xu Z., Xu B., Xu D., Tian Y., Feng W. (2012). The protective effects of riluzole on manganese-induced disruption of glutamate transporters and glutamine synthetase in the cultured astrocytes. *Biological Trace Element Research*.

[70] Pajarillo E., Johnson J., Kim J. (2018). 17*β*-estradiol and tamoxifen protect mice from manganese-induced dopaminergic neurotoxicity. *Neurotoxicology*.

[71] Li Z. C., Wang F., Li S. J. (2020). Sodium para-aminosalicylic acid reverses changes of glutamate turnover in manganese-exposed rats. *Biological Trace Element Research*.

[72] Eid T., Lee T. S. W., Patrylo P., Zaveri H. P. (2019). Astrocytes and glutamine synthetase in epileptogenesis. *Journal of Neuroscience Research*.

[73] Wedler F. C., Denman R. B. (1984). Glutamine synthetase: the major Mn(II) enzyme in mammalian brain. *Current Topics in Cellular Regulation*.

[74] Aschner M., Aschner J. L. (1991). Manganese neurotoxicity: cellular effects and blood-brain barrier transport. *Neuroscience and Biobehavioral Reviews*.

[75] Kim G., Lee H. S., Bang J. S., Kim B., Ko D., Yang M. (2015). A current review for biological monitoring of manganese with exposure, susceptibility, and response biomarkers. *Journal of Environmental Science and Health, Part C*.

[76] Huyghe D., Nakamura Y., Terunuma M. (2014). Glutamine Synthetase Stability and Subcellular Distribution in Astrocytes Are Regulated by *γ*-Aminobutyric Type B Receptors. *The Journal of Biological Chemistry*.

[77] Burton N. C., Schneider J. S., Syversen T., Guilarte T. R. (2009). Effects of chronic manganese exposure on glutamatergic and GABAergic neurotransmitter markers in the nonhuman primate brain. *Toxicological Sciences*.

[78] Erikson K. M., Dorman D. C., Fitsanakis V., Lash L. H., Aschner M. (2006). Alterations of oxidative stress biomarkers due to in utero and neonatal exposures of airborne manganese. *Biological Trace Element Research*.

[79] Erikson K. M., Dorman D. C., Lash L. H., Aschner M. (2005). Persistent alterations in biomarkers of oxidative stress resulting from combined in utero and neonatal manganese inhalation. *Biological Trace Element Research*.

[80] Chen C. J., Liao S. L. (2002). Oxidative stress involves in astrocytic alterations induced by manganese. *Experimental Neurology*.

[81] Yoo H. C., Yu Y. C., Sung Y., Han J. M. (2020). Glutamine reliance in cell metabolism. *Experimental & Molecular Medicine*.

[82] Albrecht J., Zielińska M. (2019). Exchange-mode glutamine transport across CNS cell membranes. *Neuropharmacology*.

[83] Milatovic D., Yin Z., Gupta R. C. (2007). Manganese induces oxidative impairment in cultured rat astrocytes. *Toxicological Sciences*.

[84] Sidoryk-Wecgrzynowicz M., Lee E. S., Ni M., Aschner M. (2010). Manganese-induced downregulation of astroglial glutamine transporter SNAT3 involves ubiquitin-mediated proteolytic system. *Glia*.

[85] Nissen-Meyer L. S. H., Popescu M. C., Hamdani E. H., Chaudhry F. A. (2011). Protein kinase C-mediated phosphorylation of a single serine residue on the rat glial glutamine transporter SN1 governs its membrane trafficking. *The Journal of Neuroscience*.

[86] Gavin C. E., Gunter K. K., Gunter T. E. (1990). Manganese and calcium efflux kinetics in brain mitochondria. Relevance to manganese toxicity. *Biochemical Journal*.

[87] Gavin C. E., Gunter K. K., Gunter T. E. (1999). Manganese and calcium transport in mitochondria: implications for manganese toxicity. *Neurotoxicology*.

[88] Rama Rao K. V., Norenberg M. D. (2004). Manganese Induces the Mitochondrial Permeability Transition in Cultured Astrocytes. *The Journal of Biological Chemistry*.

[89] Yin Z., Aschner J. L., dos Santos A. P., Aschner M. (2008). Mitochondrial-dependent manganese neurotoxicity in rat primary astrocyte cultures. *Brain Research*.

[90] Gonzalez L. E., Juknat A. A., Venosa A. J., Verrengia N., Kotler M. L. (2008). Manganese activates the mitochondrial apoptotic pathway in rat astrocytes by modulating the expression of proteins of the Bcl-2 family. *Neurochemistry International*.

[91] Liu Y., Barber D. S., Zhang P., Liu B. (2013). Complex ii of the mitochondrial respiratory chain is the key mediator of divalent manganese-induced hydrogen peroxide production in microglia. *Toxicological Sciences*.

[92] Ferger A. I., Campanelli L., Reimer V. (2010). Effects of mitochondrial dysfunction on the immunological properties of microglia. *Journal of Neuroinflammation*.

[93] Gorojod R. M., Alaimo A., Porte Alcon S., Saravia F., Kotler M. L. (2017). Interplay between lysosomal, mitochondrial and death receptor pathways during manganese-induced apoptosis in glial cells. *Archives of Toxicology*.

[94] Alaimo A., Gorojod R. M., Miglietta E. A., Villarreal A., Ramos A. J., Kotler M. L. (2013). Manganese induces mitochondrial dynamics impairment and apoptotic cell death: A study in human Gli36 cells. *Neuroscience Letters*.

[95] Chtourou Y., Trabelsi K., Fetoui H., Mkannez G., Kallel H., Zeghal N. (2011). Manganese induces oxidative stress, redox state unbalance and disrupts membrane bound ATPases on murine neuroblastoma cells in vitro: protective role of silymarin. *Neurochemical Research*.

[96] Rose J., Brian C., Woods J. (2017). Mitochondrial dysfunction in glial cells: implications for neuronal homeostasis and survival. *Toxicology*.

[97] Martinez-Finley E. J., Gavin C. E., Aschner M., Gunter T. E. (2013). Manganese neurotoxicity and the role of reactive oxygen species. *Free Radical Biology & Medicine*.

[98] Archibald F. S., Tyree C. (1987). Manganese poisoning and the attack of trivalent manganese upon catecholamines. *Archives of Biochemistry and Biophysics*.

[99] Barhoumi R., Faske J., Liu X., Tjalkens R. B. (2004). Manganese potentiates lipopolysaccharide-induced expression of NOS2 in C6 glioma cells through mitochondrial-dependent activation of nuclear factor kappaB. *Molecular Brain Research*.

[100] Vavougios G., Zarogiannis S. G., Doskas T. (2018). The putative interplay between DJ-1/NRF2 and dimethyl fumarate: a potentially important pharmacological target. *Multiple Sclerosis and Related Disorders*.

[101] Lee E., Yin Z., Sidoryk-Węgrzynowicz M., Jiang H., Aschner M. (2012). 15-Deoxy-*Δ*12,14-prostaglandin J2 modulates manganese-induced activation of the NF-*κ*B, Nrf 2, and PI3K pathways in astrocytes. *Free Radical Biology & Medicine*.

[102] da Silva Santos V., Bisen-Hersh E., Yu Y. (2014). Anthocyanin-rich açaí (Euterpe oleracea Mart.) extract attenuates manganese-induced oxidative stress in rat primary astrocyte cultures. *Journal of Toxicology and Environmental Health. Part A*.

[103] Exil V., Ping L., Yu Y. (2014). Activation of MAPK and FoxO by manganese (Mn) in rat neonatal primary astrocyte cultures. *PLoS One*.

[104] Giordano G., Pizzurro D., VanDeMark K., Guizzetti M., Costa L. G. (2009). Manganese inhibits the ability of astrocytes to promote neuronal differentiation. *Toxicology and Applied Pharmacology*.

[105] Yang X., Yang H., Wu F. (2018). Mn inhibits GSH synthesis via downregulation of neuronal EAAC1 and astrocytic xCT to cause oxidative damage in the striatum of mice. *Oxidative Medicine and Cellular Longevity*.

[106] Pchitskaya E., Popugaeva E., Bezprozvanny I. (2018). Calcium signaling and molecular mechanisms underlying neurodegenerative diseases. *Cell Calcium*.

[107] Guerra-Gomes S., Sousa N., Pinto L., Oliveira J. F. (2018). Functional roles of astrocyte calcium elevations: from synapses to behavior. *Frontiers in Cellular Neuroscience*.

[108] Ijomone O. M., Aluko O. M., Okoh C. O. A., Martins A. C., Aschner M. (2019). Role for calcium signaling in manganese neurotoxicity. *Journal of Trace Elements in Medicine and Biology*.

[109] Tjalkens R. B., Zoran M. J., Mohl B., Barhoumi R. (2006). Manganese suppresses ATP-dependent intercellular calcium waves in astrocyte networks through alteration of mitochondrial and endoplasmic reticulum calcium dynamics. *Brain Research*.

[110] Streifel K. M., Miller J., Mouneimne R., Tjalkens R. B. (2013). Manganese inhibits ATP-induced calcium entry through the transient receptor potential channel TRPC3 in astrocytes. *Neurotoxicology*.

[111] Hayakawa K., Esposito E., Wang X. (2016). Transfer of mitochondria from astrocytes to neurons after stroke. *Nature*.

[112] Robinson M. B., Lee M. L., DaSilva S. (2020). Glutamate transporters and mitochondria: signaling, co-compartmentalization, functional coupling, and future directions. *Neurochemical Research*.

[113] Yan D.-Y., Xu B. (2020). The role of autophagy in manganese-induced neurotoxicity. *Frontiers in Neuroscience*.

[114] Ortiz-Rodriguez A., Arevalo M. A. (2020). The contribution of astrocyte autophagy to systemic metabolism. *International Journal of Molecular Sciences*.

[115] Chu C. T. (2019). Mechanisms of selective autophagy and mitophagy: implications for neurodegenerative diseases. *Neurobiology of Disease*.

[116] Gorojod R. M., Alaimo A., Porte Alcon S., Pomilio C., Saravia F., Kotler M. L. (2015). The autophagic- lysosomal pathway determines the fate of glial cells under manganese- induced oxidative stress conditions. *Free Radical Biology & Medicine*.

[117] Gorojod R. M., Alaimo A., Porte Alcon S. (2018). Heme oxygenase-1 protects astroglia against manganese-induced oxidative injury by regulating mitochondrial quality control. *Toxicology Letters*.

[118] Zhang Z., Yan J., Bowman A. B., Bryan M. R., Singh R., Aschner M. (2020). Dysregulation of TFEB contributes to manganese-induced autophagic failure and mitochondrial dysfunction in astrocytes. *Autophagy*.

[119] Di Malta C., Cinque L., Settembre C. (2019). Transcriptional regulation of autophagy: mechanisms and diseases. *Frontiers in Cell and Development Biology*.

[120] Chen J., Su P., Luo W., Chen J. (2018). Role of LRRK2 in manganese-induced neuroinflammation and microglial autophagy. *Biochemical and Biophysical Research Communications*.

[121] Wang D., Zhang J., Jiang W. (2017). The role of NLRP3-CASP1 in inflammasome-mediated neuroinflammation and autophagy dysfunction in manganese-induced, hippocampal-dependent impairment of learning and memory ability. *Autophagy*.

[122] Porte Alcon S., Gorojod R. M., Kotler M. L. (2020). Kinetic and protective role of autophagy in manganese-exposed BV-2 cells. *Biochimica et Biophysica Acta-Molecular Cell Research*.

[123] Chang J. Y., Liu L. Z. (1999). Manganese potentiates nitric oxide production by microglia. *Molecular Brain Research*.

[124] Sarkar S., Malovic E., Jin H., Kanthasamy A., Kanthasamy A. G., Costa N. (2019). Chapter four-the role of manganese in neuroinflammation, in: M. Aschner, L.G.B.T.-a. *Role Inflamm. Environ. Neurotox*.

[125] Popichak K. A., Afzali M. F., Kirkley K. S., Tjalkens R. B. (2018). Glial-neuronal signaling mechanisms underlying the neuroinflammatory effects of manganese. *Journal of Neuroinflammation*.

[126] Kim J., Pajarillo E., Rizor A. (2019). LRRK2 kinase plays a critical role in manganese-induced inflammation and apoptosis in microglia. *PLoS One*.

[127] Zhang P., Hatter A., Liu B. (2007). Manganese chloride stimulates rat microglia to release hydrogen peroxide. *Toxicology Letters*.

[128] Kirkley K. S., Popichak K. A., Afzali M. F., Legare M. E., Tjalkens R. B. (2017). Microglia amplify inflammatory activation of astrocytes in manganese neurotoxicity. *Journal of Neuroinflammation*.

[129] Sarkar S., Rokad D., Malovic E. (2019). Manganese activates NLRP3 inflammasome signaling and propagates exosomal release of ASC in microglial cells. *Science Signaling*.

[130] Zhao X., Yin L., Wu Y. (2019). Manganese induces neuroinflammation via NF-*κ*B/ROS NLRP3 pathway in rat brain striatum and HAPI cells. *Molecular & Cellular Toxicology*.

[131] Sarkar S., Malovic E., Harischandra D. S. (2018). Manganese exposure induces neuroinflammation by impairing mitochondrial dynamics in astrocytes. *Neurotoxicology*.

[132] Liddelow S. A., Guttenplan K. A., Clarke L. E. (2017). Neurotoxic reactive astrocytes are induced by activated microglia. *Nature*.

[133] Bernaus A., Blanco S., Sevilla A. (2020). Glia crosstalk in neuroinflammatory diseases. *Frontiers in Cellular Neuroscience*.

[134] Erikson K., Aschner M. (2002). Manganese causes differential regulation of glutamate transporter (GLAST) taurine transporter and metallothionein in cultured rat astrocytes. *Neurotoxicology*.

[135] Erikson K. M., Suber R. L., Aschner M. (2002). Glutamate/aspartate transporter (GLAST), taurine transporter and metallothionein mRNA levels are differentially altered in astrocytes exposed to manganese chloride, manganese phosphate or manganese sulfate. *Neurotoxicology*.

[136] Zheng W., Zhao Q., Slavkovich V., Aschner M., Graziano J. H. (1999). Alteration of iron homeostasis following chronic exposure to manganese in rats^1^. *Brain Research*.

[137] Weber S., Dorman D. C., Lash L. H., Erikson K., Vrana K. E., Aschner M. (2002). Effects of manganese (Mn) on the developing rat brain: oxidative-stress related endpoints. *Neurotoxicology*.

[138] Dobson A. W., Weber S., Dorman D. C., Lash L. K., Erikson K. M., Aschner M. (2003). Oxidative stress is induced in the rat brain following repeated inhalation exposure to manganese sulfate. *Biological Trace Element Research*.

[139] Erikson K. M., Dorman D. C., Lash L. H., Dobson A. W., Aschner M. (2004). Airborne manganese exposure differentially affects end points of oxidative stress in an age- and sex-dependent manner. *Biological Trace Element Research*.

[140] Morello M., Zatta P., Zambenedetti P. (2007). Manganese intoxication decreases the expression of manganoproteins in the rat basal ganglia: an immunohistochemical study. *Brain Research Bulletin*.

[141] Xu B., Xu Z. F., Deng Y. (2010). Protective effects of MK-801 on manganese-induced glutamate metabolism disorder in rat striatum. *Experimental and Toxicologic Pathology*.

[142] Sidoryk-Wegrzynowicz M., Lee E., Albrecht J., Aschner M. (2009). Manganese disrupts astrocyte glutamine transporter expression and function. *Journal of Neurochemistry*.

